# Challenges and ongoing initiatives towards better integrated EU scientific advice

**DOI:** 10.3389/fmed.2025.1473346

**Published:** 2025-01-27

**Authors:** Iordanis Gravanis, Michael Berntgen, Spiros Vamvakas, Pierre Demolis, Paolo Foggi

**Affiliations:** ^1^European Medicines Agency, Amsterdam, Netherlands; ^2^Lyfjastofnun-Icelandic Medicines Agency, Reykjavík, Iceland; ^3^Agenzia Italiana del Farmaco - Italian Medicines Agency, Rome, Italy

**Keywords:** Research and Development (R&D), scientific advice, clinical trials, medical devices, innovative combination products, health technology assessment, new EU pharmaceutical legislation

## Abstract

Scientific advice is the main avenue for clarification of EU regulators’ scientific evidence requirements during medicines development. There are multiple avenues for seeking scientific advice in the EU with partially overlapping scope which creates room for divergence and contradictions; simplification and better integration among them could help harmonize EU regulators’ requirements. Interaction with other decision makers providing advice along the lifecycle of medicines and other healthcare solutions reduces development uncertainties. The proposal for a new EU pharmaceutical legislation solidifies existing advice mechanisms and creates new avenues for enhanced integration of development support.

## 1 Introduction

Scientific advice refers to several different interactions with European Union (EU) regulatory authorities during medicines development aimed at clarifying regulators’ scientific evidence requirements applicable during the development, most notably prior to the initiation of clinical trials, and/or for the eventual market approval (marketing authorization). These requirements are often detailed in international and/or EU-specific scientific guidelines, but scientific advice may provide clarity in situations where there is little or outdated guidance. Scientific advice may also help clarify how existing guidance should be applied in a case-specific context. It constitutes the core and main form of regulatory support to medicines developers towards optimization of scientific evidence generation to support approval of new medicines, new uses of existing medicines and/or other major (usually manufacturing) post-authorization changes.^1^ Other forms of regulatory development support include both formal (e.g., orphan designation,^2^ pediatric medicines support,^3^ priority medicines-PRIME designation)^4^ and informal^5^ interactions which are outside the scope of this manuscript.

The EU medicines development support ecosystem comprises regulators and other, parallel or subsequent, decision-makers and has been criticized as being too fragmented, sometimes leading to conflicting advice and recommendations. The proposal for the new pharmaceutical legislation published by the European Commission in April 2023^6^ attempts to simplify regulatory decision-making at European Medicines Agency (EMA) level, but the proposal is still in the legislative process and, more importantly, it does not address the complexity outside the remit of the EMA in the wider ecosystem. Main challenges preventing integrated and hence more coherent EU scientific advice will be analyzed in the following, with a focus on clinical development where the fragmentation notably occurs.

## 2 Policy options and implications

### 2.1 The current scope of scientific advice and its proposed amendment in the draft legal proposal for reform of the EU pharmaceutical legislation

According to the EU legislation,^7^ scientific advice is about ‘advising undertakings on the conduct of the various tests and trials necessary to demonstrate the quality, safety and efficacy of medicinal products for human use and of veterinary medicinal products’. In the absence of a legal definition, undertakings could be understood as medicine developers at large, mainly pharmaceutical companies and, less commonly, other entities developing new or existing medicines. In practice, scientific questions on any aspect of medicines development and any part of the dossier supporting a clinical trial or marketing authorization application fall within the scope of scientific advice.

Scientific advice focuses on prospective development planning aspects and refrains from pre-assessment of the actual data produced in the course of development. The assessment of such data takes place at marketing authorization application stage, when an authorization decision is made focusing on the balance of benefits and risks and going well beyond experiment and study design aspects into the evidence that ultimately supports the conclusion on benefits, risks, uncertainties around them and necessary post-authorization follow-up. Although focused on future development plans, scientific advice cannot ignore but is instead informed by early exploratory evidence which is critical for scientific advice at any stage of development. This is best exemplified in the case of tailored scientific advice for biosimilars,^8^ where reduced non-clinical and clinical development programs can be proposed based on promising, rather extensive analytical comparability data. Review of such data informs the advice given, but it is without prejudice to their eventual detailed assessment during the marketing authorization application.

On the other hand, scientific advice formally assesses evidence in the case of qualification of novel methodologies (QoNM). Such qualification implies regulatory acceptability of novel methodologies for use in medicines development within a specific context in which they have been validated.^9^ Examples of such methodologies include novel biomarkers to be used for enrichment of patient populations in early clinical trials or novel patient reported outcomes (PROs) to be used as secondary endpoints in confirmatory clinical trials. Scientific advice can be sought in early stages of method development on the proposed validation plan, but can also be used for the assessment of the evidence leading to regulatory qualification. Once it has been concluded that the proposed method can be qualified for a well-defined context of use, a qualification opinion is published^10^ and subjected to public consultation before being finalized.

The revised Regulation^11^ included in the European Commission proposal for reform of the EU pharmaceutical legislation expands the legal provisions for scientific advice (articles 58 and 59), albeit for the most part formalizing practices already in place or mirroring other recent pieces of legislation. Notable changes, the majority of which address the EU development support fragmentation, include:

1) Contrasting ‘undertakings’ to not-for-profit entities as scientific advice applicants. This implies that undertakings are to be understood as pharmaceutical companies and generally as for-profit entities in contrast to purely academic applicants, learned societies and other not-for-profit entities. The new Regulation further foresees fee reductions and waivers for not-for-profit entities which the new EMA fee Regulation (EU) 2024/568,^12^ applicable as of January 2025, has already put in place
2) leveraging of clinical trial and medical device expertise from national competent authorities to support centralized scientific advice, as necessary
3) consultation of other authorities and public bodies, as applicable
4) parallel consultations with health technology assessment (HTA) bodies and with the expert panels for medical devices
5) publication of high-level information from scientific advice at the time of marketing authorization.

### 2.2 Options for seeking scientific advice from regulators in the EU and associated challenges in medicines development

There are multiple avenues for applicants to seek advice from EU regulators^13^ and these include national, simultaneous national (SNSA) and centralized (also called EMA, SAWP, or CHMP) scientific advice. This is in contrast to the US system with the existence of the centralized Food and Drug Administration (FDA) solely responsible for a multitude of meeting types^14^ intended to support both clinical trial^15^^,^^16^ and marketing authorization applications.^17^^,^^18^ Underpinning this complexity, which understandably creates challenges for navigating the EU regulatory development support landscape, is a compartmentalization of remits between the EMA and National Competent Authorities (NCAs) for medicines with the former being responsible for EU-wide marketing authorizations while the latter are responsible for any clinical trial and national marketing authorizations.

The scopes of national, simultaneous national and centralized scientific advice are partially overlapping, each one offering advice on any product, any aspect of the dossier supporting subsequent regulatory applications and at any stage of the medicine’s development. However, as scientific advice is sought in preparation for subsequent regulatory decisions, each advice option is more commonly used in different stages of medicines development depending on the remit of the regulatory decision-maker providing the advice. The scientific advice strategy is the developer’s choice and may entail national advice and SNSA more frequently in earlier stages of development in order to support subsequent clinical trial applications whilst centralized advice is sought most commonly ahead of phase 3 clinical development in order to clarify marketing authorization requirements. SNSA was launched in 2020 in the form of a pilot in two phases to date and, while the scope is generally identical to single national scientific advice, it offers the possibility for applicants to get advice on the same set of questions and data package from different National Competent Authorities (NCAs) of EU member states within a single procedure.^19^

Provision of centralized scientific advice is the task of the Scientific Advice Working Party (SAWP) of the Committee of Medicinal Products for Human use (CHMP). The committee itself is responsible for producing scientific opinions which form the basis of EU marketing authorization decisions by the European Commission. The SAWP comprises experts from the European Medicines Regulatory Network (EMRN)^20^ representing different types of expertise involved in medicines development including members from relevant EMA working parties and the majority of EMA scientific committees^21^ as well as academic experts. This composition ensures provision of best advice possible and consistency between scientific advice and subsequent regulatory decision-making of different types (maintenance of orphan designation, pediatric investigation plan (PIP) agreement, authorization of advanced therapy medicines and adequacy of post-authorization follow-up and pharmacovigilance plans). It also allows the identification of regulatory guidance gaps, e.g., in case of novel technologies or evolving treatment landscapes, so that existing guidance can be updated or new guidelines can be developed, which a task of EMA working parties other than the SAWP.

On the other hand, the multitude of EMA working parties and especially of scientific committees creates challenges for the agile and coherent provision of regulatory development support. This is, e.g., obvious in the case of pediatric medicines development as both SAWP and Pediatric Committee (PDCO) guide on prospective development plans albeit with different remits. The proposal for the new pharmaceutical legislation foresees refocusing on two main committees for human medicines with a view to simplification of regulatory decision-making and increased efficiency and harmonization. Retention of expertise of outgoing committees would be enabled through alternative means such as a pool of experts to be consulted. The legislative proposal therefore creates the opportunity for more agile decision-making through involvement of subject matter experts in each case without the need for committee-level endorsement and formal opinion adoption.

The proposed new legislation maintains the SAWP as a working party of the CHMP with the sole remit of providing scientific advice and hence also maintains the separation of scientific advice from subsequent regulatory evaluation. The principle separation between individuals in prominent roles during early advice and later assessment, respectively, has been recommended to prevent any perceived conflict of opinion whilst recognizing the need to balance such principle against allowing to employ necessary scientific expertise.^22^ Obviously, in depth knowledge of the product and the development is scientifically relevant for the assessment of the marketing authorization application and any post-authorization lifecycle changes of the medicinal product. Applying such principle on those individuals in prominent roles is feasible but requires careful management and sufficient capacity to not add to the existing resource constraints in the EU medicines regulatory network.^23^

The authorization of clinical trials at national level is another major challenge for medicines development in the EU and lack of harmonization of clinical trial application requirements across EU member states has repeatedly been identified as a major obstacle towards conduct of multi-national clinical trials in the EU. The clinical trials regulation, applicable since January 2022, is aimed at ensuring that the EU offers an attractive and favorable environment for carrying out clinical research on a large scale, with high standards of public transparency and safety for clinical trial participants.^24^

The Accelerating Clinical Trials in the EU (ACT-EU)^25^^,^^26^ initiative, also launched in January 2022, builds on the clinical trials regulation and aims to transform how clinical trials are initiated, designed and run, in order to further promote the development of high quality, safe and effective medicines, and to better integrate clinical research in the European health system. The ACT-EU Priority Action 7 focuses on scientific advice ahead of clinical trial applications and two pilots were launched in June 2024^27^^,^^28^ with the aim to reinforce regulators’ advice ahead of clinical trial applications, as follows:

1) Consolidated scientific advice on clinical trial and marketing authorization requirements by the SAWP with the involvement of the Clinical Trials Coordination Group (CTCG):^29^^,^^30^ this follows the centralized scientific advice process with at least one of the two SAWP coordinators identified from the member states expected to coordinate the assessment of the subsequent clinical trial application. Individual member state comments concerning clinical trial requirements additional to the consolidated advice (if inevitable) are also communicated in the SAWP final advice letter. This initiative is in line with the European Commission proposal for the new pharmaceutical legislation which foresees leveraging of clinical trial expertise from national competent authorities to support centralized scientific advice.
2) Consolidated technical and regulatory advice (not scientific advice) by the CTCG, called pre-CTA advice:^31^ this uses the SNSA avenue for submission but follows a shortened timeline, as intended to address technical and regulatory issues towards a smooth clinical trial application (CTA).

### 2.3 Parallel scientific advice with other decision-makers

Marketing authorization is a critical but not the final decision towards patient and market access for medicines. Health technology assessment (HTA)^32^ informs subsequent reimbursement and pricing decisions taken at EU member state level. The EMA has been collaborating with HTA bodies^33^ through the European Network for Health Technology Assessment (EUnetHTA)^34^ since 2010 towards both provision of parallel scientific advice, started in 2012, and towards building synergies between regulatory evaluation and the HTA. The Regulation (EU) 2021/2282 on Health Technology Assessment^35^ foresees joint scientific consultations (JSCs) between HTA bodies to be carried out by the HTA Coordination Group and optionally in parallel with the scientific advice process of the EMA. Well-established processes of parallel scientific advice between EMA and HTA bodies have been used to prepare for implementation and establishment of the new parallel consultation process under the new HTA regulation.

Moreover, use of medicinal products is becoming increasingly linked to medical devices which can be integral to or co-packaged with the medicine or used separately from it but support (*in vitro* diagnostic) or dictate (companion diagnostic) its use. Medical devices and in vitro diagnostics are regulated in the EU via respective Regulations (Regulation (EU) 2017/745^36^ and Regulation (EU) 2017/746).^37^ Both Regulations foresee scientific advice from expert panels^38^ established by them. However, scientific advice from the expert panels is not available to manufacturers of *in vitro* (including companion) diagnostics [such advice is legally available only to the European Commission and the Medical Devices Coordination Group (MDCG)]. Moreover, scientific advice from the expert panels is restricted to high-risk medical devices which are primarily used on their own and not in combination with medicines. Finally, expert panels comprise clinical experts who can only advise on clinical, but not quality, development aspects.

Although clearly of value within its remit, scientific advice from the medical device expert panels cannot address the major device-related issues of current and future medicines development. These issues relate to combination products, i.e., medicines used in combination with medical devices or *in vitro* diagnostics. Most notable examples of innovative combination products are targeted therapies given to biomarker-defined populations for which a companion diagnostic is used to ascertain the status of the biomarker and hence identify patients eligible (or non-eligible) for the targeted therapy. Such combination products are already commonplace, mainly in hematology/oncology but also other therapeutic areas. The issues in the development of combination products stem primarily from the integrated conduct of the clinical investigation for the medical device or the performance study for the *in vitro* diagnostic with the clinical trial for the medicine in the combination. Different frameworks and regulators govern the approval and conduct of clinical investigations, performance studies and clinical trials following different timelines and requirements. Moreover, there is still no EU-coordinated process for multi-national clinical investigation or performance study approval, while coordinated review of clinical trial applications is already taking place since January 2022 following the go-live of the EU Clinical Trials Information System (CTIS).^39^

In many EU member states, medicinal products and medical devices/*in vitro* diagnostics are regulated by the same NCA and the scope of both national scientific advice and SNSA also covers combination products as long as these combination products fall within the remit of the NCA or NCAs participating in the SNSA pilot and their scientific-regulatory advice services. Similarly, questions on medical devices/*in vitro* diagnostics used in combination products are routinely being addressed in centralized scientific advice having access to medical device expertise in NCAs represented in the SAWP.

However, these advice options do not address the needs in terms of scope and capacity while some medical device decision-makers such as notified bodies are legally constrained from providing advice during device development. In order to address these issues lying at the interface between the Regulations on clinical trials, medical devices and *in vitro* diagnostics, the European Commission has launched the COMBINE project^40^ in an attempt to harmonize the approval of combined studies, i.e., studies integrating a medical device clinical investigation or *in vitro* diagnostic performance study within a clinical trial.

Much as the focus in the EU is currently on coordination and harmonization among Member States in both areas of clinical trials and medical devices/*in vitro* diagnostics, there would also be benefits from further exchanges on scientific advice beyond the EU borders with international medicines regulators. The EMA and the US FDA have been operating a process of parallel scientific advice^41^ since 2005 with modest uptake by medicines developers to date (1). The reasons may relate to logistical challenges of applicants dealing with two regulatory agencies in parallel in a process that involves additional meetings and effort. Scientific advice interactions between developers and EMA or FDA are relatively short and simple and more cross-border exchanges certainly increase procedural complexity, although they clearly add value and create opportunities for international harmonization of regulators’ scientific evidence expectations.

## 3 Actionable recommendations and conclusions

To be meaningful, development support and guidance for scientific evidence generation need to evolve. Scientific advice is the pillar for obtaining feedback from EU regulators on the development plan. Several initiatives have been taken and pilots have been initiated to strengthen the ecosystem; the proposal for a revised pharmaceutical legislation builds on these experiences. It is recognized that better coordination is needed within clinical trial approval processes to improve consistency and predictability, particularly for studies combining medicinal products with medical devices or *in vitro* diagnostics, and make the EU competitive again in the area of clinical research. Closer links with medical device regulators at national level could help optimize existing and/or develop new scientific advice mechanisms for combination products which are becoming the norm, especially in therapeutic areas like oncology. More intense collaboration of medicines regulators with HTA bodies could improve patient access to new medicines. Critical expertise needs to be retained and remain accessible for the future fewer EMA committees and working parties, while their mode of operation should also adapt to their enhanced responsibilities. Finally, simplification and integration of the multiple EU scientific advice avenues and ensuring capacity of European NCAs to provide EU-level work may help ease resource constraints in the EU medicines regulatory system while making it simpler for medicines developers to seek regulators’ advice.

## Footnotes

^1^Scientific advice and protocol assistance | European Medicines Agency (europa.eu).

^2^Orphan designation: research and development | European Medicines Agency (europa.eu).

^3^Paediatric medicines: Overview | European Medicines Agency (europa.eu).

^4^PRIME: priority medicines | European Medicines Agency (europa.eu).

^5^Supporting innovation | European Medicines Agency (europa.eu) | advice mechanisms.

^6^Reform of the EU pharmaceutical legislation – European Commission (europa.eu).

^7^Article 57(n) of Regulation (EC) No 726/2004 laying down Union procedures for the authorisation and supervision of medicinal products for human use and establishing a European Medicines Agency.

^8^Scientific advice and protocol assistance | European Medicines Agency (europa.eu).

^9^Qualification of novel methodologies for medicine development | European Medicines Agency (europa.eu).

^10^Opinions and letters of support on the qualification of novel methodologies for medicine development | European Medicines Agency (europa.eu).

^11^Proposal for a Regulation laying down Union procedures for the authorisation and supervision of medicinal products for human use and establishing rules governing the European Medicines Agency.

^12^Regulation (EU) 2024/568 on fees and charges payable to the European Medicines Agency (europa.eu).

^13^Advice on medicines for Human use in the EMRN (europa.eu).

^14^Formal Meetings between the FDA and Sponsors or Applicants of PDUFA Products.

^15^Investigational New Drug (IND) Application | FDA.

^16^Investigational New Drug Applications (INDs) for CBER-Regulated Products | FDA.

^17^New Drug Application (NDA) | FDA.

^18^Biologics License Applications (BLA) Process (CBER) | FDA.

^19^Heads of Medicines Agencies (HMA) EU Innovation Network (EU-IN), section on Simultaneous National Scientific Advice (SNSA).

^20^European medicines regulatory network | European Medicines Agency (europa.eu).

^21^Committees, working parties and other groups | European Medicines Agency (europa.eu).

^22^Decision in strategic inquiry OI/7/2017/KR on how the European Medicines Agency engages with medicine developers in the period leading up to applications for authorisations to market new medicines in the EU | Decision | European Ombudsman.

^23^Handling competing interests | European Medicines Agency (EMA).

^24^Regulation (EU) No 536/2014 on clinical trials on medicinal products for human use

^25^Accelerating clinical trials in the EU (europa.eu).

^26^Accelerating Clinical Trials in the EU (ACT EU) | European Medicines Agency (europa.eu).

^27^Scientific advice – European Union (europa.eu).

^28^Scientific advice and protocol assistance | European Medicines Agency (europa.eu) | Scientific advice on clinical trials.

^29^Heads of Medicines Agencies: Clinical Trials Coordination Group (hma.eu).

^30^Guidance for applicants SAWP CTCG pilot on scientific advice (europa.eu).

^31^Guidance for applicants Pre-CTA advice pilot_final (europa.eu).

^32^Health Technology Assessment-Overview-European Commission (europa.eu).

^33^Health technology assessment bodies | European Medicines Agency (europa.eu).

^34^www.eunethta.eu

^35^Regulation (EU) 2021/2282 on health technology assessment.

^36^Regulation (EU) 2017/745 7 on medical devices.

^37^Regulation (EU) 2017/746 on in vitro diagnostic medical devices.

^38^Medical device expert panels | European Medicines Agency (EMA).

^39^Clinical Trials Information System | European Medicines Agency (europa.eu).

^40^Combined studies - European Commission (europa.eu).

^41^Scientific advice and protocol assistance | European Medicines Agency (europa.eu) | Parallel scientific advice with the United States.
